# Identification of a unique humoral immune signature of Sudan ebolavirus persistence in human survivors

**DOI:** 10.21203/rs.3.rs-9601480/v1

**Published:** 2026-05-26

**Authors:** Shuangyi Bai, Haruna Muwonge, Carolyne Nasimiyu, Barnabas Bakamutumaho, Cynthia Ombok, Sinem Ulusan, Peter James Elyanu, Moses L. Joloba, Robert F. Breiman, Isaac Ssewanyana, Susan Nabadda, Julius Lutwama, Misaki Wayengera, Bruce Kirenga, Henry Kyobe Bosa, M. Kariuki Njenga, Bronwyn M. Gunn

**Affiliations:** 1 Paul G. Allen School for Global Health, Washington State University. Pullman, WA, USA.; 2 Makerere University Medical School. Kampala, Uganda.; 3 Washington State University Global Health-Kenya. Nairobi, Kenya.; 4 Interdisciplinary Consortium on Epidemic Research/ Uganda Virus Research Institute. Entebbe, Uganda.; 5 Baylor-Uganda. Kampala, Uganda; 6 Rollins School of Public Health, Emory University. Atlanta, GA, USA; 7 Uganda Central Public Health Laboratories. Kampala, Uganda.; 8 Ministry of Health Uganda. Kampala, Uganda.

## Abstract

Since finding that long-term persistence of ebolavirus RNA exists in a subset of human survivors for up to 5 years post infection in several organs, viral reactivation has been implicated in recurrence of acute disease among ebolavirus disease survivors and linked to small disease outbreaks. Thus, identifying correlates of ebolavirus persistence are critical to the long-term care of survivors and outbreak management. We analyzed the humoral immune response using a comprehensive systems serology approach in 86 the 87 survivors (98.8%) of the 2022–2023 Sudan ebolavirus (SUDV) outbreak in Uganda. Across the survivors, 55% of eligible survivors (20 of 36 survivors) were found to have persistence of viral RNA in either semen or breastmilk for up to 6 months following initial infection, whereas the remaining 45% tested negative (16 of 36 survivors). We found an elevated, unique, and sustained humoral immune signature associated with persistence of viral RNA in SUDV survivors and specifically have identified 4 humoral immune features that together predicted persistence: glycoprotein-specific antibody dependent cellular phagocytosis [ADCP], nucleoprotein-specific IgG2, nucleoprotein-specific IgA1, and VP40-specific IgM. Moreover, analysis of the 4 features in the remaining 50 SUDV survivors who were ineligible for semen or breastmilk sampling, predicted an additional 17 survivors with humoral immune responses consistent with viral RNA persistence survivors. We also find that antibodies against the VP40 (matrix protein), were associated with faster clearance of persistent viral RNA. Thus, a subset of humoral immune responses could be important for monitoring and clearing viral persistence in ebolavirus disease survivors.

## INTRODUCTION

Orthoebolaviruses, including Zaire ebolavirus (EBOV) and Sudan ebolavirus (SUDV), have caused explosive outbreaks of severe disease in multiple regions of Africa and is associated with high mortality and long-term clinical sequelae for surviving individuals. Uganda has experienced seven outbreaks of SUDV, with the most recent in early 2025, affecting 14 people with 4 deaths. A larger outbreak in 2022–2023 resulted in 142 infections with 55 deaths^1^. The increasing frequency of these outbreaks in the last 5 years has highlighted a persistent and growing threat that orthoebolaviruses present to the region. In addition to the morbidity and mortality associated with acute infection, survivors of ebolavirus disease (EVD) face a myriad of long-term complications including debilitating sequelae and persistence of viral RNA in a subset of survivors^2^.

EBOV RNA can persist in semen, vitreous humor, breast milk, cerebrospinal fluid in EVD survivors for months to years^3–5^. Viral RNA is estimated to persist in the semen in 90% male survivors during the first 3 months in convalescence, dropping to 25% by 9 months, and is estimated to be present in 1–3% of survivors at 2 years^6^. While the presence of viral RNA does not necessarily prove infectious potential in a majority of survivors, virus recrudescence presumably from a survivor of the 2014–2016 epidemic has been posited to be responsible for the small 2021 outbreak in Guinea^4^, and transmission of the virus sexually and via breastmilk have been documented^7,8^. To date, only routine testing of semen from consenting male survivors or lactating female survivors are performed, as sampling of ocular vitreous humor or cerebral spinal fluid are invasive procedures and not routinely feasible. Thus, non-lactating female and child survivors are rarely tested but could be at risk for persistence of viral RNA beyond the convalescence period.

Antibodies against ebolavirus are protective against EBOV and SUDV infections in animal models, providing prophylactic and therapeutic protection against challenge via both potent neutralizing activity and innate immune effector functions through the antibody Fc domain^9–11^. Human efficacy trials of monoclonal antibodies led to the approval of a monotherapy as well as a monoclonal antibody cocktail for treatment of acute human infection. Moreover, antibodies are the correlate of protection for the WHO pre-qualified live vaccine against Ebola virus, Erevbo (rVSV-ZEBOV)^12^. Long-term humoral immune responses are induced in survivors of ebolavirus infection^13–15^, and fluctuations in antibody responses observed in survivors has been posited to be a reflection of recrudescence of viral antigen that stimulates a rise in ebolavirus-specific antibodies^16^. Thus, ebolavirus-specific antibodies may be an effective biomarker of persistence of viral RNA that could be measured in all survivors. However, whether a specific antibody response is associated with clearance of persistent viral RNA is unknown.

Here, we characterize the humoral immune response of 86 survivors of the 2022 SUDV outbreak in Uganda, starting at 2–3 months post infection and continued at quarterly intervals through 15 months. At each follow-up, semen or breast milk from 36 eligible and consenting adult survivors were tested for SUDV RNA by PCR, with 20 (55%) testing positive at least one follow-up. Using a comprehensive systems serology approach that profile Fc-mediated antibody effectors functions, we characterized the longitudinal humoral antibody response against SUDV of survivors and a group of community controls and determined if humoral immunity was altered in the context of persistent viral RNA.

## METHODS AND MATERIALS

### Human serum samples.

Serum samples from 86 (98.8%) survivors of the SUDV outbreak in Uganda (September-December 2022) were collected at quarterly timepoints beginning two to three months after discharge from the Ebola Treatment Unit (ETU). The demographics of the survivors enrolled in our study are shown in Table 1. In addition, 192 community controls were collected in October 2023 according to approved study protocol from the School of Biomedical Sciences Research Ethics Committee at Makerere University (Approval #SBS-2022–243) and the Uganda National Council of Science and Technology (Approval #HS2618ES). Additional administrative approvals were obtained from the Uganda Ministry of Health and participating health facilities. Written informed consent was obtained from all participants prior to enrollment. Serum samples were heat-inactivated at 56°C for 30 minutes prior to testing.

### Cell lines and reagents.

Vero E6 cells (ATCC CRL-1586) were maintained in D10 medium (DMEM supplemented with 10% FBS, 1% penicillin–streptomycin and 1% L-glutamine) at 37 °C in 5% CO_2_. THP-1 monocytes (ATCC TIB-202) were maintained in D10 medium (DMEM supplemented with 10% FBS, 1% penicillin–streptomycin, 1% L-glutamine, and 55μM beta-mercaptoethanol). HL60 cells (ATCC CCL-240) were maintained in Iscove's Modified Dulbecco's Medium supplemented with 10% FBS, 1% penicillin–streptomycin and 1% L-glutamine and differentiated for 5 days in the presence of 1.3% DMSO. NK92 cells expressing CD16 (158V; ATCC PTA-6967) were maintained in alpha-MEM (2mM L-glutamine and 1.5g/L of sodium bicarbonate) supplemented with 12.5% FBS, 12.5% horse serum, 20μM folic acid, 200μM inositol, 100μM beta-mercapoethanol, and 0.2mg/ml of IL-2.

### Multiplexed-Luminex based antibody subclassing.

GP is the major surface protein on both the virion and the infected cell^17^; sGP is secreted from infected cells, acting as an immunomodulator and antibody decoy^18^; NP is highly immunogenic and plays a critical role in viral replication^19^; and VP40 is the matrix protein that is involved in viral budding and egress from infected cells^20^. Recombinant SUDV GP, sGP, VP40 (IBT Bioservices), and NP (Sino Biologics) were coupled to MagPlex beads (Luminex) according to previously published protocols^21^.

Samples were diluted to 1:500 (Total IgG, IgG1, IgM) or 1:25 (IgG2, IgG3, IgG4, total IgA, IgA1, and IgA2) and incubated with antigen-coupled beads for 2h. Following bead washing, antibody subclasses (IgG1, IgG2, IgG3, IgG4) and isotypes (IgM, IgA1, IgA2) were detected using PE-labeled secondary antibodies (0.65μg/ml; Southern Biotech). The geometric mean fluorescent intensity (gMFI) of 30 beads/region was recorded and analyzed on an Intelliflex instrument (Luminex).

### Seropositivity threshold.

To establish a seropositivity threshold, we analyzed 24 randomly selected serum samples from adults (without history of being exposed to ebolavirus) from neighboring Kenya (n=16) to consider potential cross-reactivity from other viruses or environmental factors common in East Africa, and the United States that were collected in 2020 (n=8), prior to the SUDV outbreak in Uganda. To limit the potential for false positives, the seropositivity threshold was conservatively defined as the mean of these 24 controls + 5X the standard deviation.

### Neutralization assay.

Vero E6 cells were seeded at 10,000 cells per well in 384-well flat-bottom microplates (Thermo Scientific, cat. # 464718) in 50 μl of D10 medium and incubated 24h. On Day 2, serial three-fold dilutions of serum (starting at 1:20; eight dilutions) were prepared in D10 medium. Recombinant vesicular stomatitis virus pseudotyped with the SUDV Gulu glycoprotein (rVSV–SUDV-Gulu-GP) and encoding a luciferase reporter was obtained from IBT Bioservices (cat. # 1002–002). An equal volume of pseudovirus (160 RLU μl^−1^) was added to each dilution and incubated for 1 h at 37 °C. Cell culture medium was removed by gently flicking the plate, after which 25 μl of the virus–antibody mixture was added to each well. Plates were centrifuged at 1,200 × g for 1 h at 4 °C and subsequently incubated for 24 h at 37 °C in 5% CO_2_. Luciferase activity was measured by adding 30 μl per well of Bright-Glo luciferase substrate (Promega, cat. #. E2620) and reading luminescence on a Tecan Spark luminometer (Life Sciences). Percent neutralization was calculated by normalizing pseudovirus-only wells to 0% neutralization and cell-only wells to 100% neutralization, as described previously^22^. Neutralization curves were fitted using nonlinear regression (“log(inhibitor) vs. normalized response—variable slope”) in GraphPad Prism v10 to derive 50% inhibitory dilution (ID_50_) values. Each assay plate included a neutralizing monoclonal antibody as a positive control and either COVID-19–positive sera from a US-based cohort or assay medium as a negative control.

### Antibody-dependent phagocytosis (ADNP and ADCP).

Biotinylated SUDV GP or VP40 was conjugated to red Neutravidin beads (ThermoFisher) and incubated with samples for 2h prior to adding HL-60 cells differentiated with 1.3% DMSO for 5 days blood (5×10^4^ cells/well) for 2h (ADNP) or adding THP-1 cells at 2.5×10^4^ cells/well for 18h (ADCP). Uptake of antibody-bead complexes by cells was determined by analysis on a Cytek Aurora spectral flow cytometer, and a phagocytic score was determined: (% bead positive^+^ cells)*(gMFI red beads^+^)/10,000. The gating strategy is shown in **Figure S1.**

### Antibody-mediated complement deposition (ADCD).

Biotinylated SUDV GP-coated red Neutravidin beads (ThermoFisher) were incubated with heat-inactivated samples. Guinea pig complement (Cedarlane Labs) diluted in veronal buffer containing calcium and magnesium (Boston Bioproducts) was incubated with antibody-bead complexes for 20min, and C3 deposition onto beads was detected using an anti-guinea pig C3 antibody (MP Biomedicals) and analyzed on a Cytek Aurora spectral flow cytometer. The gating strategy is shown in **Figure S1**.

### Antibody-dependent NK cell degranulation.

ELISA plates were coated with SUDV GP-antigen (300ng/well). Wells were washed, blocked, and incubated with samples for 2h prior to adding NK-92 cells expressing CD16 (5×10^4^ cells/well) for 5h with brefeldinA, GolgiStop, and anti-CD107a (Biolegend). Intracellular cytokine staining to detect IFNγ and TNFα (Biolegend) was performed using Fix/Perm (Biolegend), and cells were analyzed on a Cytek Aurora spectral flow cytometer. The gating strategy is shown in **Figure S1**.

### Discriminant analysis.

A regularized discriminant analysis was performed using a stepwise variable selection method using antibody measures at each timepoint (eg 2–6 months post-infection) to classify survivors with and without persistent viral RNA in JMP Pro 16 (SAS).

### Calculation of the combined humoral immune score.

A combined score based on selected features was calculated using the following formula: ((Feature 1 value)*(Feature 2 value)*(Feature 3 value)*(Feature 4 value))^(1/4).

### ROC analysis.

Combined humoral immune scores for survivors with and without persistent virus was used to generate an ROC using Prism 10 software. The Youden’s index was calculated using the following formula: (sensitivity + specificity)-1.

### Statistical analysis.

Univariate analyses were performed using Prism 10 software. Kruskal-Wallis with Dunn’s multiple correction test or Mann-Whitney with Bonferroni correction were used to determine statistical significance between groups, and paired Wilcoxan-rank was used to compare changes in immunity between timepoints in the same individual.

## RESULTS

### Antibody profile of SUDV survivors 2–15 months post-infection.

**Figure 1.**
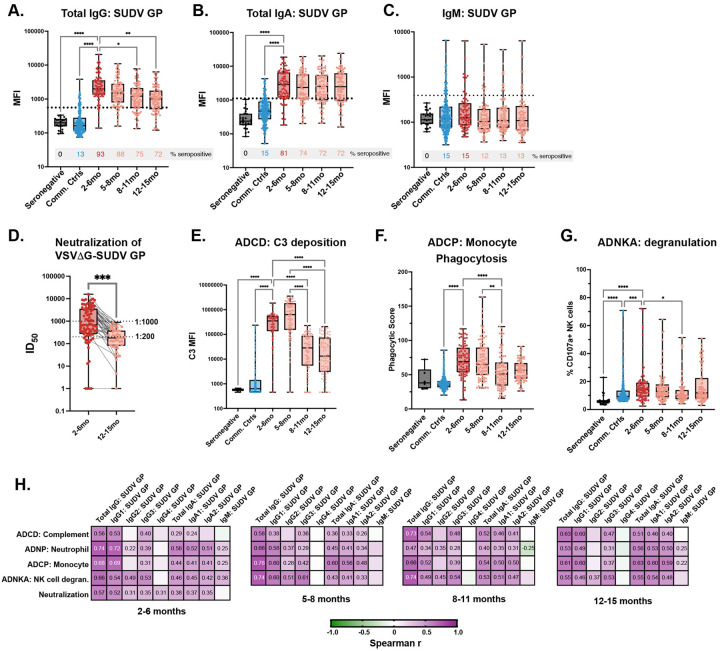
Humoral immune profiles of SUDV survivors across 2–15 months post-infection. (A-C) Levels of GP-specific total IgG (A), GP-specific total IgA (B), and GP-specific IgM (C) were measured in seronegative controls (n=24), Uganda community controls (n=192), and SUDV survivors across 4 timepoints. The dashed lines indicate threshold of seropositivity for each measure. Kruskal-Wallis test with Dunn’s correction for multiple comparisons. (D-G) The inhibitory dose 50 (ID_50_) of VSVDG-SUDV GP pseudovirus was measured for the indicated groups (D) and the ability of GP-specific antibodies to induce complement activation (E), phagocytosis by monocytes (F) or NK cell degranulation (G) were measured for the indicated groups. Kruskal-Wallis test with Dunn’s correction for multiple comparisons. The dotted lines in (D) represent the ID_50_ of 1:200 and 1:1000, as indicated to the right of the graph. The lines connect survivor’s ID_50_ values at the two timepoints. H. Spearman correlation analyses were performed between levels of GP-specific IgG subclasses, IgA subclasses, total IgG, total IgA, or IgM with indicated antibody-mediated effector functions. The spearman rho values of statistically significant associations at indicated timepoints are shown (not corrected for multiple comparisons), and are colored where purple is a positive correlation, and green is a negative correlation.

To characterize the humoral immune responses in SUDV survivors, Uganda community controls, and confirmed ebolavirus seronegative controls from neighboring Kenya and the US, we applied a systems serology approach to determine the quantity and quality of SUDV-specific antibodies, including measurement of IgG, IgA, IgM, and IgG and IgA subclasses against four different SUDV antigens: glycoprotein (GP), soluble glycoprotein (sGP), nucleoprotein (NP) and viral protein 40 (VP40). Across the survivors, 81 (93%) were IgG seropositive against GP at enrollment, which declined to 72% by 12–15 months post-infection (Figure 1A). This temporal pattern was similar for IgG1, IgG2, and IgG3 against all the four viral antigens (**Figure S2**). In contrast to the decline in IgG, levels of IgA antibodies against GP, sGP, and NP remained relatively steady between enrollment and 12–15 months post-infection (Figure 1B, **Figure S4**). Strikingly, we did not observe VP40-specific IgA responses, (**Figure S4**), highlighting antibody profile differences against SUDV antigens. Most survivors were IgM seronegative against GP, with only 15% of survivors having detectable IgM responses at enrollment (Figure 1C), consistent with reduced detection of IgM after 12 weeks post-infection. As expected, none of the seronegative controls had IgM or IgG antibodies detected. However, approximately 13–15% of the Uganda community controls had detectable SUDV-specific IgG, IgA, or IgM. As samples from the community controls were collected in October 2023, these responses may reflect exposure to SUDV during the outbreak or asymptomatic infection.

As qualitative features of GP-specific antibodies are critical to protection against EBOV^9,10,23^, we next measured antibody-mediated effector function of SUDV GP-specific antibodies including neutralization of a VSV-ΔG SUDV GP pseudovirus, and induction of Fc-mediated phagocytosis by monocytes (ADCP), neutrophils (ADNP), activation of NK cells (ADNKA), and deposition of the central complement component C3 (ADCD). Strong neutralizing activity of the VSV-ΔG SUDV GP pseudovirus, defined as ID_50_ greater than 1:200, was observed in >80% of survivors 2–3 months after infection, with 45% of survivors having an ID_50_ >1:1000. At 12–15 months post-infection, 46% of tested survivors (n=41) maintained an anti-GP ID_50_ titers >1:200 (Figure 1D). The ability of GP-specific antibodies to activate complement (Figure 1E) and induce monocyte-mediated phagocytosis (Figure 1F) was observed through 5–8 months post infection but declined by 12–15 months. Similar results were observed for GP-specific activation of NK cells (Figure 1G, **Figure S5**), and induction of neutrophil phagocytosis (**Figure S5**). We found that levels of GP-specific total IgG, IgG1, IgG3, total IgA, IgA1, and IgA2 significantly correlated with all antibody functions across the timepoints measured (Figure 1H), indicating a coordinated humoral immune response in SUDV survivors.

### Higher viral load in acute disease associated with viral RNA persistence among survivors.

Sustained detection of SUDV-specific RNA in semen or breastmilk has previously been reported in a subset of survivors from the 2022 SUDV outbreak in Uganda for 3 to 7 months post-infection^24^. Of the 86 SUDV survivors, 42 were eligible for semen or breastmilk testing for viral RNA persistence. Of those eligible, 36 consented and 20 (55.6%) of them tested positive for SUDV RNA persistence. The number of days from admission to the Ebola Treatment Unit (ETU) to the last positive PCR result after infection was calculated for each survivor and was used as a metric of duration of viral RNA persistence. These survivors remained SUDV RNA positive for a median of 131.5 days (range: 84 to 210 days, IQR = 83.3).

We investigated whether parameters of acute illness were associated with viral RNA persistence, including disease severity at viral load at time of admission into the ETU. Acute illness severity was classified by the non-mutually exclusive categorization as follows: Mild (fever, vomiting, diarrhea, loss of appetite, abdominal pain); moderate (chest pain, muscle pain, joint pain, headache, cough, sore throat); and severe (jaundice, red eye, coma, confusion, bleeding). Across the 86 survivors analyzed here, 45 (52.3%) were classified as having had severe illness, 39 (45.3%) were classified as having moderate illness, and 2 (2.3%) had mild illness. Survivors who were tested for the presence of persistent SUDV RNA experienced either severe or moderate disease during acute infection (Table 2), yet there was no statistically significant differences in severity of disease between those with and without viral RNA persistence (p=0.3). However, survivors with persistent viral RNA spent longer within the ETU than survivors without persistent viral RNA (Table 2). The length of ETU stay was elevated in the persistent RNA survivors irrespective of acute disease severity (i.e. moderate or severe disease severity) compared to survivors without persistent viral RNA, suggesting that disease symptom severity alone is insufficient to explain increased ETU stay duration.

**Figure 2.**
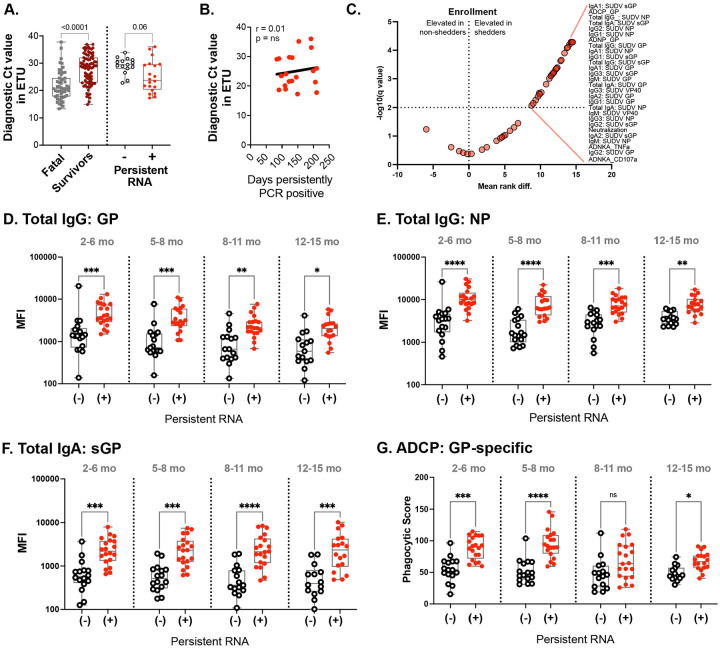
Correlates of viral persistence in SUDV survivors. A. The Ct value of the confirmatory PCR performed at admittance to the Ebola Treatment Unit during acute infection was compared between those with fatal outcomes (grey squares) and all survivors (crimson circles), and between survivors with (red circles) and without persistent (open circles) viral RNA detected in semen/breastmilk during convalescence. p values were determined by Kruskal-Wallis test with Dunn’s correction for multiple comparisons. B. Spearman correlation was performed between Ct value and days persistently RNA positive in survivors with persistent viral RNA. C. Volcano plot of a multiple Mann-Whitney tests across all SUDV-associated antibody features at the enrollment timepoint (2–6 months post-infection) between survivors with and without persistent viral RNA. The dashed line on the y-axis (log10 q value = 2) indicates a q-value <0.01 (corrected for multiple comparisons). The statistically significant features are indicated on graph, with features to the right are elevated in survivors with persistent viral RNA compared to those without. D-G. Univariate plots of the top four antibody features that were elevated in survivors with persistent viral RNA compared to those without across the indicated timepoints. (D) GP-specific total IgG; (E) NP-specific total IgG; (F) sGP-specific total IgA; (G) GP-specific Antibody-dependent cellular phagocytosis by monocytes (ADCP). Kruskal-Wallis test with Dunn’s correction for multiple comparisons.

To investigate impact of viral load at ETU admission on survival and viral RNA persistence, we correlated admission Ct values with survival and persistence of viral RNA in semen or breast milk. Overall, survivors had higher Ct values (lower viral load) than fatal cases, and survivors with persistent SUDV RNA had lower Ct values at admission when compared to those negative for virus persistence (Figure 2A). These findings indicate that higher viral load at admission increased the likelihood of viral RNA persistence among survivors. However, the range of Ct values amongst those with persistent SUDV RNA varied widely (Ct values 17.21–36), perhaps reflecting differences in acute disease stage at admission. Importantly, we did not observe a correlation between admission Ct value and duration of viral RNA persistence in convalescence (Figure 2B).

### Several humoral immune features are elevated in survivors with persistent SUDV RNA.

We performed a multiple Mann-Whitney comparison of SUDV-specific antibody levels at the enrollment timepoint (2–3 months post-infection) between survivors with and without persistent SUDV RNA (Figure 2C, **Figure S6**). The data are summarized as a volcano plot, where the adjusted q-value (after correction for multiple comparisons) for each measure is shown on the Y axis, and values that exceed −log10 q value ≥ 2 (q value ≤ 0.01) were considered statistically significant. The mean difference in rank of survivors with persistent RNA vs survivors without persistent RNA is shown on the X-axis, where positive mean rank differences indicate that the respective antibody response is elevated in those with RNA detected compared to those without (Figure 2C).

Of the 44 antibody features included in the analysis, 28 measures were significantly elevated in survivors with persistent viral RNA, indicating a significantly altered humoral immune response against SUDV antigens in the context of viral RNA persistence. These measures spanned all viral antigens tested, and included all IgG and IgA subclasses, except for IgG4 (Table 3). A subset of antibody responses continued to be elevated in survivors with persistent RNA compared to those without, across multiple follow-up visits spanning 1-year post-infection (Table 3). Notably, IgA (including both IgA1 and IgA2) responses against GP, sGP, and NP remained elevated through to 15 months post-infection in survivors with persistent RNA. Univariate plots of the top features that were elevated in survivors with persistence (GP-specific total IgG [Figure 2D], NP-specific total IgG [Figure 2E], sGP-specific total IgA [Figure 2F], and ADCP activity [Figure 2G]) are shown over time.

### Distinct humoral profiles can predict RNA persistence.

**Figure 3.**
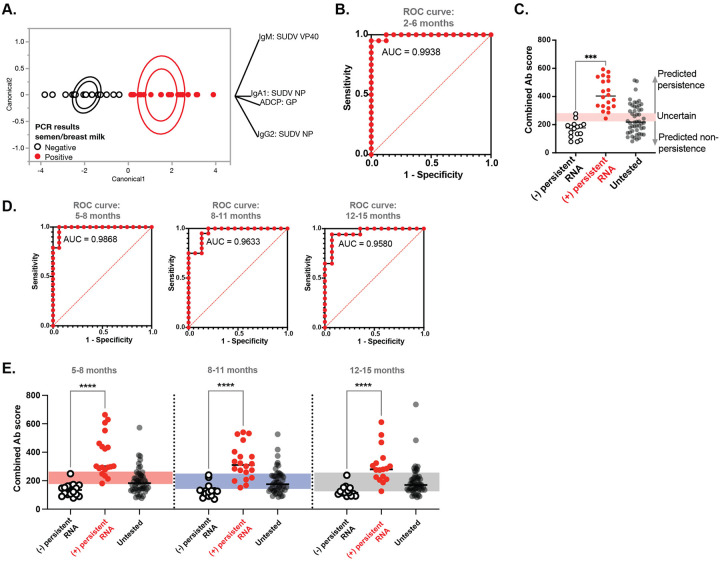
Distinct humoral profiles can predict RNA persistence. **A.** A regularized discriminant analysis was performed using a stepwise variable selection method using antibody measures at the enrollment timepoint (2–6 months post-infection) to classify survivors with and without persistent viral RNA. Four features were selected by the model are indicated in the loadings biplot. A combined score using these four features was calculated using the following formula: (Feature 1 value)*(Feature 2 value)*(Feature 3 value)*(Feature 4 value)^(1/4). **B.** A combined score using the four features identified in (A) was calculated for each survivor at the enrollment timepoint using the following formula: (Feature 1 value)*(Feature 2 value)*(Feature 3 value)*(Feature 4 value)^(1/4). The resulting combined antibody score was then used to generate a ROC curve for classification of survivors with and without persistent viral RNA. The AUC of the ROC curve is indicated on the plot. **C.** Comparison of the combined antibody scores between survivors without persistent RNA (open circle), survivors with persistent RNA (Red circle), and survivors who were unable to be tested for the presence of persistent RNA (grey circles). The shaded region indicates the bounds of the upper and lower antibody scores from the ROC curve for either 100% sensitivity (upper) or 100% specificity (lower). **D.** The combined antibody score for the survivors with and without persistent RNA at the later timepoints (5–8 months, 8–11 months, and 12–15 months) were used to generate ROC curves for each timepoint. The AUC for each is indicated on the plot. **E.** The combined antibody scores between survivors without persistent RNA (open circles), survivors with persistent RNA (red circles), and survivors who were unable to be tested for the presence of persistent RNA (grey circles) for each timepoint. The shaded region indicates the bounds of the upper and lower antibody scores from the ROC curve for either 100% sensitivity (upper) or 100% specificity (lower) for the different timepoints (red shaded 5–8 months; blue shaded 8–11 months; grey shaded 12–15 months)

We used these data to define the minimum number of features needed to predict viral RNA persistence at enrollment using a discriminant analysis with variable selection (Figure 3A). Subsequently, we generated a composite humoral immune score for each timepoint based on the features selected to establish a cutoff above which survivors were categorized as the “persistence” category and below would fall into the “no persistence” category (Figure 3B). At the enrollment time point (2–3 months post-infection), we identified 4 immune features (GP-specific antibody dependent cellular phagocytosis [ADCP], NP-specific IgG2, NP-specific IgA1, and VP40-specific IgM) that produced a ROC curve with an AUC of 0.9938. We then calculated the Youden’s index from the ROC curve and selected maximum sensitivity (100%) with the highest Youden’s index to determine a threshold humoral immune score that could be applied on enrolled survivors to predict prolonged viral RNA persistence. Using this approach, we estimated that 17 of the other 50 untested survivors had immune features consistent with survivors with persistent RNA (Figure 3C).

We further assessed the value of the composite immune score at the follow-up time points to classify persistence. The ROC curve AUC for responses measured at 5 months post-infection was 0.9868, 8 months post-infection was 0.9633, and 12 months post-infection was 0.958 (Figure 3D). Across these time points, 6 of the 17 untested survivors had the immune feature signature consistent with persistence across all 4 timepoints above the Youden’s index with the maximum specificity (100%) to ensure no false positives (Figure 3E).

### Level of IgG against VP40 is associated with virus clearance.

**Figure 4.**
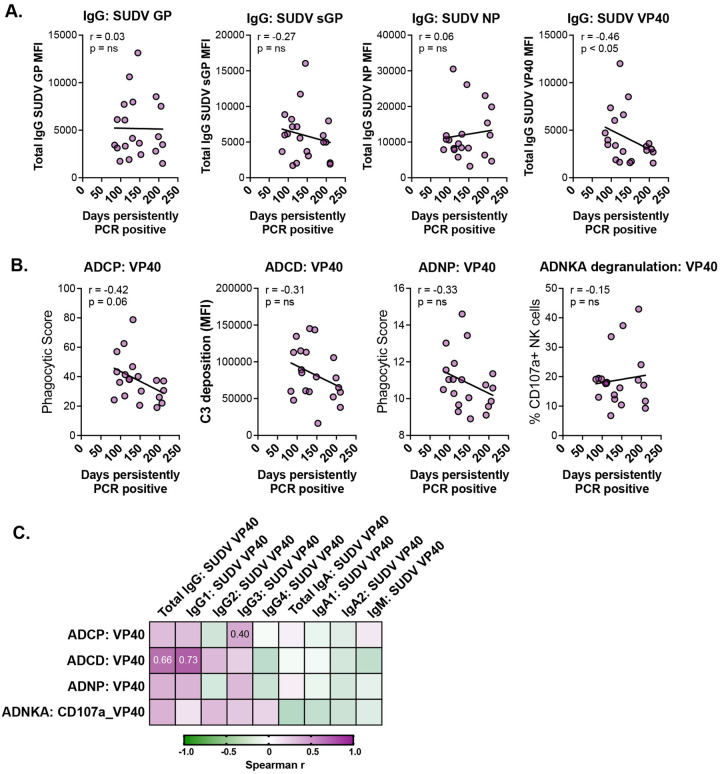
VP40-specific responses are associated with accelerated clearance of persistent viral RNA. **A.** Spearman correlation analyses were performed between levels of total IgG specific for GP, sGP, NP, and VP40 and days persistently RNA positive in survivors with persistent viral RNA. The spearman rho and p value are indicated on each graph. **B.** Spearman correlation analyses were performed between levels of VP40-specific antibody-mediated effector functions (antibody-dependent cellular phagocytosis by monocytes [ADCP], antibody-dependent complement deposition [ADCD], antibody-dependent neutrophil phagocytosis [ADNP], or antibody-dependent NK cell degranulation [ADNKA] and days persistently RNA positive in survivors with persistent viral RNA. The spearman rho and p value are indicated on each graph. **C.** Spearman correlation heatmap of VP40-specific IgG subclasses, IgA subclasses, and IgM with VP40-specific antibody-mediated effector functions. The spearman rho values of statistically significant associations at indicated timepoints are shown (not corrected for multiple comparisons), and are colored where purple is a positive correlation, and green is a negative correlation.

To determine if any antibody feature was associated with long term viral RNA persistence, we used the number of days from ETU admission positive PCR to last positive PCR result after infection for each patient to perform spearman correlation analysis with each humoral immune feature. Across the SUDV-specific measures collected at enrollment, we found that total IgG and IgG1 levels against VP40 were negatively correlated with days of persistent shedding (Figure 4A, **Figure S7**), indicating that higher VP40-specific IgG1 levels tracked with accelerated clearance of viral RNA from semen/breast milk. To determine if VP40-specific antibodies could mediate innate immune effector functions, we measured ADCP, ADNP, ADNKA, and ADCD against VP40-coated targets, and then determined if a specific effector response tracked with accelerated clearance of viral RNA. We found that ADCP responses were negatively associated with duration of viral RNA persistence, while ADCD and ADNP had a similar trend but not statistically significant (Figure 4B). Interestingly, while ADCP activity was positively correlated with VP40-specific total IgG or IgG1 levels, these associations did not reach statistical significance (Figure 4C), possibly due to the small sample size. However, ADCP activity was significantly associated with VP40-specific IgG3 potentially suggesting that additional qualitative features of VP40-specific IgG3 may be independently associated with accelerated viral persistence.

## DISCUSSION

Ebolavirus persistence and recrudescence have been demonstrated in multiple clinical, epidemiological, and genomic studies, and implicated in causing acute, sometimes fatal disease in survivors, and transmission that seeds new EVD clusters^4,25–27^. Ebolavirus persistence has been demonstrated in ocular fluid, semen, central nervous system including brain parenchyma and cerebrospinal fluid, and breast milk^5,6,27–30^. While persistence is well studied in survivors infected with EBOV, there is a paucity of data on persistence in SUDV, and more importantly on the association between host immune response and persistence of any of the ebolavirus strains.

Here, we used a well-characterized SUDV survivor cohort to define key antibody profiles associated with viral persistence, identifying 4 immune markers that can predict viral persistence among EVD survivors. As many immune-privileged sites are not easily and safely accessible, surveillance of persistent viral RNA has been limited to analyses of semen and breastmilk in eligible survivors. Thus, non-lactating women and children are typically not screened for persistence, but other host tissue reservoirs of ebolavirus persistence may exist in these survivors. When we assessed immune profiles of 50 untested survivors, over one-third (n=17) had immune signatures consistent with viral RNA persistence. Importantly, the immune signature was sustained in 6 of these 17 survivors (35%) through the 15 months post infection timepoints that we evaluated, suggesting that these humoral immune markers can be used to monitor EVD survivors for viral persistence, potentially negating the need for invasive sampling and testing of tissues for the virus.

The underlying mechanisms for viral RNA persistence in a subset of EVD survivors are not well understood. Although using a limited sample size, we observed that higher viral load (lower Ct values) during acute infection had a direct association with incidence of persistence^6^, but no necessarily the duration of persistence. Previous studies have posited that intermittent spikes of ebolavirus-specific IgG during convalescence result from recrudescence of virus^16^. Our finding of elevated SUDV-specific humoral immune responses in persistence survivors over 1.5 years post-infection supports this position. Importantly, while overall SUDV-specific IgG levels decline over time, we find that IgG4 levels against GP, sGP, and NP rose over time in SUDV survivors, although not statistically significant overall as a group, but GP and sGP-specific IgG4 significantly increased within individuals over time (**Figure S3**). While RNA persistence in specific compartments was not a significant predictor of IgG4 magnitude, the longitudinal increase in IgG4 across the cohort suggests that SUDV-specific B-cell maturation and class-switching continue well into convalescence, potentially driven by prolonged antigen exposure in a subset of individuals.

Early (<6 months post infection) humoral immune responses in human survivors of EBOV survivors are characterized by highly polyfunctional responses against the GP^31^, and similarly, the humoral immune response that we have characterized here indicates that a majority of SUDV survivors develop polyfunctional antibody responses against both the GP and VP40. While GP-specific IgG levels declined somewhat over time, we observed consistent levels of GP-specific IgA in SUDV survivors. While EBOV-specific IgA responses in EBOV survivors have been described that last for at least 1-year post-infection^32^, the sustained IgA seroprevalence across survivors is not clear. We have previously shown that GP-specific IgA can mediate antibody-dependent neutrophil phagocytosis^31^, and ADNP has been linked to vaccine-mediated protection in NHPs^33^. SUDV GP-specific IgA levels correlated with all the Fc-mediated effector functions we measured here yet may be more of a reflection of the development of a well-coordinated antibody response in survivors rather than directly mediating functional activity in all immune cells. The role of IgA in long-term immunity against ebolaviruses is still unclear, but the longevity of the IgA response observed here may warrant further analysis of this antibody isotype in the future.

Ultimately, clearance of infectious virus (suggested by persistent RNA) is needed to ensure long-term safety and health of survivors. Interestingly, we find that the duration of persistent viral RNA was inversely correlated with levels of VP40-specific IgG and with ADCP activity of VP40-coated targets. These data suggest that antibodies targeting VP40 may be worth pursuing as therapeutic candidates to accelerate clearance of persistent viral RNA. Therapeutic targeting of VP40 has been proposed during acute infection given the role of VP40 in viral budding^34,35^, and detailed mapping of the humoral immune response in an EVD survivor showed development of durable and high affinity VP40-specific IgG and IgA that preceded decline of clinical symptoms^32^. Beyond binding to a subset of FcRs^36^, the functions of VP40-specific antibodies have not been well studied, but our data indicate that these antibodies can mediate multiple effector functions and may be an important component of long-term immunity for ebolaviruses.

Our study has several limitations that must be acknowledged. The sample size of our cohort was limited to the number of survivors for the 2022–2023 outbreak. We enrolled and sampled 86 of the 87 survivors (98.8%) of this outbreak, and sampling of semen and breastmilk was performed for all eligible and consenting survivors. However, despite the small sample size, we were able to define a four-parameter humoral immune signature of persistence of viral RNA among survivors. While we cannot ascertain the predictive potential of the four-parameter humoral immune signatures in survivors, our findings provide a foundation for follow-up studies using future SUDV survivors such as the recent 10 SUDV survivors from a 2025 SUDV outbreak in Uganda that are now part of our cohort.

In summary, our study highlights the potential for humoral immune responses during convalescence to be used as a biomarker for viral persistence in human ebolavirus survivors. Moreover, our results highlight that antibodies against non-GP viral antigens, such as VP40, track with accelerated clearance of viral RNA, and thus may be an opportunity for therapeutic intervention.

## Supplementary Files

This is a list of supplementary files associated with this preprint. Click to download.

- SUDVsupplementaryfigurescombined.pdf

**Figure S1.** The gating strategies for ADNP (A), ADCP (B), ADCD (C) and ADNKA (D) are shown for representative SUDV GP seronegative and seropositive samples as indicated.

**Figure S2.** Levels of GP-specific IgG1, IgG2, IgG3, IgG4 (A), sGP-specific IgG1, IgG2, IgG3, IgG4 (B), NP-specific IgG1, IgG2, IgG3, IgG4 (C) and VP40-specific IgG1, IgG2, IgG3, IgG4 (D) were measured in seronegative controls (n=24), Uganda community controls (n=192), and SUDV survivors across 4 timepoints. Kruskal-Wallis test with Dunn’s correction for multiple comparisons.

**Figure S3.** The GP- and sGP-specific IgG4 responses for each survivor is plotted longitunally across the four timepoints (2–15 months). Mixed-effects analysis with Holm-Sidak’s multiple comparison test was used to determine if later timepoints were significantly different from the enrollment (2–6 month) timepoint.

**Figure S4.** Levels of total IgG (A), total IgA (B), and IgM (C) were measured in seronegative controls (n=24), Uganda community controls (n=192), and SUDV survivors across 4 timepoints. Kruskal-Wallis test with Dunn’s correction for multiple comparisons.

**Figure S5.** The ability of GP-specific antibodies to induce antibody-mediated neutrophil phagocytosis and antibody-mediated NK cell secretion of TNFα were measured for the indicated groups. Kruskal-Wallis test with Dunn’s correction for multiple comparisons.

**Figure S6.** Volcano plot of a multiple Mann-Whitney tests across all SUDV-associated antibody features at the 5–8 months post-infection (A), 8–11 months post-infection (B), and 12–15 months post-infection (C) between survivors with and without persistent viral RNA. The dashed line on the y-axis (log10 q value = 1.3) indicates a q-value <0.05 (corrected for multiple comparisons). The statistically significant features are indicated on graph, with features to the right are elevated in survivors with persistent viral RNA compared to those without.

**Figure S7.** Spearman correlation analyses were performed between levels of VP40-specific IgG1, IgG2, IgG3, IgG4 at 2–6 months post-infection and days persistently RNA positive in survivors with persistent viral RNA. The spearman rho and p value are indicated on each graph.

## Figures and Tables

**Table 1.** Demographic characteristics of the SUDV survivors included in this study. The age, sex, and occupation of all SUDV survivors (n=87), or survivors with (n=20) and without persistent viral RNA (n=16) are shown. P values compare means between survivors with and without persistent viral RNA by Mann-Whitney test for age; Fisher’s exact test was used to determine statistical significance between male/female distribution in survivors with and without persistent viral RNA.

	All	Persistent	Non-persistent	p value
n	87	20	16	
**Age**				
Mean (SD)	29.8 (13.05)	29.32 (9.061)	32.62 (7.375)	0.336
Median (IQR)	29.65 (15.96)	29.18 (15.91)	30.31 (10.31)	
		
<10	6 (6.9%)	0 (0%)	0 (0%)	N/A
10–19	11 (12.6%)	3 (15%)	1 (6.25%)	
20–39	55 (63.2%)	15 (75%)	12 (75%)	
40–49	10 (11.5%)	2 (10%)	3 (18.75%)	
>50	5 (5.7%)	0 (0%)	0 (0%)	
				
**Sex**				
Male (%)	56 (64.4%)	15 (75%)	16 (100%)	0.0527
Female (%)	31 (35.6%)	5 (25%)	0 (0%)	
				
**Occupation**				
HCW (%)	8 (9.2%)	3 (15%)	4 (25%)	N/A
Professional/skilled labour (%)	7 (8%)	2 (10%)	1 (6.25%)	
Unskilled labour (%)	8 (9.2%)	2 (10%)	0 (0%)	
Farmer (%)	32 (36.8%)	10 (50%)	4 (25%)	
Student (%)	10 (11.5%)	1 (5%)	2 (12.5%)	
Other/NA (%)	22 (25.3%)	2 (10%)	5 (31.25%)	

**Table 2.** Acute EVD parameters of the SUDV survivors included in this study. Acute EVD parameters of SUDV survivors (n=87), or survivors with and without persistent viral RNA are shown, including the date range of illness confirmation, disease symptom severity, and the number of days spent in the ETU. Fisher’s exact test was used to determine statistical significance in disease symptom severity (moderate vs severe) in survivors with and without persistent viral RNA. P values of mean of days spent in ETU between survivors with and without persistent viral RNA that experienced moderate or severe symptoms was determined by Kruskal-Wallis test with Dunn’s correction for multiple comparisons.

		All	Persistent	Non-persistent	p value
n		87	20	16	N/A
**Date range of illness confirmation**					
		9/20/22–11/15/2022	9/22/22–11/10/2022	9/23/22–10/28/22	N/A
**Severity of acute disease**					
Mild		3	0	0	
Moderate		39	8	10	0.3145
Severe		45	12	6	
**Number of days in ETU**					
Mean (SD)		12.39 (7.95)	17.42 (7.94)	9.69 (9.53)	<.001
Median (IQR)		10 (9)	18 (9)	7 (4)	
		
Moderate	Mean (SD)	12.27 (9.92)	18.38 (11.13)	9.7 (11.66)	p<0.05
	Median (IQR)	9 (10)	17.5 (9.25)	5.5 (5.75)	
Severe	Mean (SD)	12.85 (5.77)	16.73 (5.04)	9.67 (5.28)	p=0.08
	Median (IQR)	12 (8.5)	18 (10)	8.5 (5.25)	

**Table 3.** q values of antibody measures between shedding and non-shedding SUDV survivors. All measures with significant q values (q<0.05) were elevated in survivors with persistent viral RNA compared to survivors without viral RNA.

Isotype	Antigen	2–6 mo.	5–8 mo.	8–11 mo.	12–15 mo.
Total IgA	GP	0.00049	0.00033	0.00094	0.00106
IgA1	GP	0.00036	0.00016	0.00094	0.00106
IgA2	GP	0.00057	0.00016	0.00186	0.00064
Total IgG	GP	0.00008	0.00007	0.00068	0.00135
IgG1	GP	0.00061	0.00106	ns	ns
IgG2	GP	0.00621	0.00124	0.00588	ns
IgG3	GP	ns	0.00501	ns	ns
IgG4	GP	ns	ns	ns	ns
IgM	GP	0.00040	0.00164	ns	0.00396
Total IgA	sGP	0.00005	0.00002	0.00002	0.00054
IgA1	sGP	0.00005	0.00002	0.00002	0.00064
IgA2	sGP	0.00294	0.00164	0.00186	0.00318
Total IgG	sGP	0.00021	0.00002	0.00000	0.00002
IgG1	sGP	0.00021	0.00002	0.00000	0.00003
IgG2	sGP	0.00246	0.00124	0.00189	0.00949
IgG3	sGP	0.00038	0.00002	0.00002	0.00064
IgG4	sGP	ns	ns	ns	ns
IgM	sGP	ns	ns	ns	ns
Total IgA	NP	0.00066	0.00026	0.00094	0.00294
IgA1	NP	0.00012	0.00016	0.00047	0.00172
IgA2	NP	ns	ns	ns	ns
Total IgG	NP	0.00005	0.00001	0.00002	0.00029
IgG1	NP	0.00008	0.00001	0.00004	0.00029
IgG2	NP	0.00005	0.00001	0.00000	0.00029
IgG3	NP	0.00127	0.00036	0.00186	ns
IgG4	NP	ns	ns	ns	ns
IgM	NP	0.00349	ns	ns	ns
Total IgA	VP40	ns	ns	ns	ns
IgA1	VP40	ns	ns	ns	ns
IgA2	VP40	ns	ns	ns	ns
Total IgG	VP40	ns	ns	ns	ns
IgG1	VP40	ns	ns	ns	ns
IgG2	VP40	ns	ns	0.00189	ns
IgG3	VP40	0.00053	0.00020	0.00i86	0.00688
IgG4	VP40	ns	ns	ns	ns
IgM	VP40	0.00081	ns	ns	0.00640
Function	Antigen	2–6 mo.	5–8 mo.	8–11 mo.	12–15 mo.
Neutralization	GP	0.0032	n.d.	n.d.	n.d.
ADCP	GP	0.0001	0.00002	0.0251	0.0009
ADNP	GP	0.0001	0.0011	ns	ns
ADCD	GP	ns	0.0006	0.0021	0.0160
ADNKA: CD017a	GP	0.0087	0.0003	0.0061	0.0361
ADNKA: TNFa	GP	0.0057	0.0002	0.0330	0.0568

ns = not significant, q<0.05

n.d. = not determined
